# Disappointment and adherence among parents of newborns allocated to the control group: a qualitative study of a randomized clinical trial

**DOI:** 10.1186/1745-6215-15-126

**Published:** 2014-04-15

**Authors:** Sandra Meinich Petersen, Vibeke Zoffmann, Jesper Kjærgaard, Lone Graff Steensballe, Gorm Greisen

**Affiliations:** 1 Department of Neonatology, the Danish National Hospital – “Rigshospitalet”, Blegdamsvej 9, 2100 København Ø, Copenhagen, Denmark; 2 Steno Diabetes Center, Niels Steensens Vej 2, 2820 Gentofte, Denmark; 3 NK LMS, Oslo Universitetssykehus HF, Ullevål, Postboks 4956, Nydalen, 0424 Oslo, Norway; 4 The Child and Adolescent Clinic, the Danish National Hospital – “Rigshospitalet”, Blegdamsvej 9, 2100 København Ø, Copenhagen, Denmark

**Keywords:** Adherence, Allocation, Altruism, Control group, Clinical trial, Disappointment, Motives, Newborns, Parents, Randomized controlled trial

## Abstract

**Background:**

When a child participates in a clinical trial, informed consent has to be given by the parents. Parental motives for participation are complex, but the hope of getting a new and better treatment for the child is important. We wondered how parents react when their child is allocated to the control group of a randomized controlled trial, and how it will affect their future engagement in the trial.

**Methods:**

We included parents of newborns randomized to the control arm in the Danish Calmette study at Rigshospitalet in Copenhagen. The Calmette study is a randomized clinical trial investigating the non-specific effects of early BCG-vaccine to healthy neonates. Randomization is performed immediately after birth and parents are not blinded to the allocation. We set up a semi-structured focus group with six parents from four families. Afterwards we telephone-interviewed another 19 mothers to achieve saturation. Thematic analysis was used to identify themes across the data sets.

**Results:**

The parents reported good understanding of the randomization process. Their most common reaction to allocation was disappointment, though relief was also seen. A model of reactions to being allocated to the control group was developed based on the participants’ different positions along two continuities from ‘Our participation in trial is not important’ to ‘Our participation in trial is important’, and ‘Vaccine not important to us’ to ‘Vaccine important to us’. Four very disappointed families had thought of getting the vaccine elsewhere, and one had actually had their child vaccinated. All parents involved in the focus group and the telephone interviews wanted to participate in the follow-ups planned for the Calmette study.

**Conclusions:**

This study identified an almost universal experience of disappointment among parents of newborns who were randomized to the control group, but also a broad expression of understanding and accepting the idea of randomization. The trial staff might use the model of reactions in understanding the parents’ disappointment and in this way support their motives for participation. A generalized version might be applicable across randomized controlled trials at large.

**Trial registration:**

The Calmette study is registered in EudraCT (https://eudract.ema.europa.eu/) with trial number 2010-021979-85.

## Background

Before patients participate in a clinical trial they must give signed informed consent stating that they understand and accept the terms of the trial [1]. For randomized controlled trials (RCTs) this also means understanding and accepting that there is a probability, most often 50%, of being allocated to the control group thus receiving no treatment, placebo, or standard treatment. In general, patients often struggle to understand the study design of an RCT; the concept of randomization and its implications can be difficult to understand and accept [2].

Several studies have investigated patients’ motives for participating in clinical trials and found that altruism along with the hope of personal gain were the dominant motives among the patients [3-5]. The hope of personal gain is probably tied up with being allocated to the treatment group, rather than the control group [6]. If patients in general hope to receive the active treatment, it is reasonable to assume that they will feel disappointed if allocated to the control group, but few studies have actually addressed this issue. In a smoking cessation trial expressions of disappointment were common and drop-out rates tended to be higher in the intervention group compared to the control group, although not statistically so; the most dissatisfied were those who did not understand the study design [7]. In contrast, in a trial in rheumatic diseases, patients who were lost to follow-up more often stated personal issues as the reason rather than dissatisfaction with the trial [8].

When the patient is a child, informed consent has to be given by the parents, which places the parents in a difficult situation since their role is to protect and care for the child. When being asked to participate in a randomized trial, parents do not only have to balance the risks against the potential benefits as if they were to be research subjects themselves, but furthermore they have to make the ‘right’ decision on behalf of their child in order to feel like good parents [9]. Their motives for letting the child participate are therefore likely to be complex. The hope of getting a new and better treatment is important [2,10], but parents also see the decision about trial participation as an important responsibility and find it appropriate that they are asked for their consent, even though it remains a difficult decision [11].

Parents’ reactions to and understanding of their child’s allocation in an RCT were studied qualitatively at the end of a trial where severely ill newborns were allocated to conventional treatment or to transferal for extracorporeal membrane oxygenation [2]. Interviews were carried out before the results of the RCT were known. Parents of children in the control group expressed disappointment because continuing standard intensive care in a situation of life-threatening illness was viewed as doing nothing. A later study was conducted with a different group of parents from the same trial after the parents had been told that the RCT had shown extracorporeal membrane oxygenation to be effective and to save lives [12]. After this new information, the parents of the intervention group objected to the concept of randomization since they thought that the intervention had saved their child, whereas the parents of the ‘standard care’ group had experienced that their child had survived without intervention and consequently were less critical of the concept of randomization.

We wondered how parents react when their child is allocated to the control group in a less critical context. Do they feel disappointed or relieved, and how do they cope with their emotional response? We also wondered if their way of coping influences their motivation towards the continued participation in the trial.

To answer these questions we set up a qualitative study including parents of children who were allocated to the control group of the Calmette study at Rigshospitalet, Copenhagen.

### The Calmette study

The Danish Calmette trial is an ongoing randomized controlled clinical trial, taking place from 2012 to 2015 in three hospitals in Denmark, and aiming to enroll 4,300 newborns from the general population and randomize them to BCG (Calmette) vaccination versus no intervention. The BCG vaccine was withdrawn from the Danish Child Vaccination Program in 1982 when protection against tuberculosis was no longer relevant since the incidence of tuberculosis in Denmark had become low. The adherence to the Danish Child Vaccination Program is generally good with, for example, 91% to 94% of the newborn population immunized with the diphtheria, tetanus, pertussis, polio, and haemophilus influenza vaccine at ages 3 months, 5 months, and 12 months. The Calmette-study investigates the potential positive non-specific effects of early BCG vaccine in high-income settings. The hypothesis tested in the Calmette-study is if the BCG vaccine, besides the specific protection against tuberculosis, causes a general, non-specific modulation of the immune system that could reduce infectious diseases in the first years of life as well as the incidence of atopic dermatitis, asthma, and allergies [13], as recently documented in low-income settings in West Africa [14]. The BCG vaccine has been widely used globally since 1921 and side effects are well known and described [15]; the risk of severe side effects is small [16]. After vaccination, a characteristic sore usually develops at the injection site lasting two to four months, resulting in a small scar [17]. Therefore, the parents in the Calmette-study were not blinded to the allocation as no suitable placebo mimicking the sore and scar exists.

Recruitment for the Calmette study took place during pregnancy, but randomization was delayed until after the infant was born. Written information about the study was sent to the parents by mail in the beginning of the third trimester of the pregnancy, including an information sheet explaining the background, aims, course, side effects, and potential benefits and risks along with information about the general rights of participants in biomedical trials (Additional file 1). A couple of weeks later, the family received a phone call from a member of the research team in order to elaborate on the information sheet and to discuss participation in the trial. The parents were given the opportunity to consider participation and get in contact with trial staff as many times as they needed before giving their informed consent. If the parents decided to participate, they signed the consent form that had been sent along with the written information, and mailed it to the trial staff. Written informed consent could also be given at the hospital. A few hours after delivery, the midwife in charge or the Calmette study research staff performed the computerized randomization, and children allocated to the active group were vaccinated immediately whereas children allocated to the control group received no intervention. All personnel performing randomization and the intradermal BCG-injection were specially trained in the procedures. If, for some reason, randomization was postponed, the child could still be randomized (and immunized if allocated to the active group) within 7 days of birth.

## Methods

**Figure 1:**
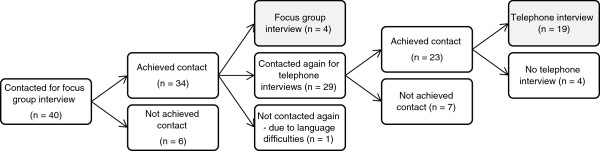
Flow chart of the recruitment process.

The subjects were parents of children participating in the Danish Calmette study who had been allocated to the control group. Hence, we did not contact parents of children allocated to the intervention group. We planned a focus group with eight families, but only four families took part and we subsequently conducted semi-structured telephone interviews with an additional group of 19 mothers based on the preliminarily identified themes of the focus group [18]. The recruitment process (Figure 1) is described below.

### Focus group

We contacted the parents of the children, but we only had the mothers’ telephone numbers, not the fathers’. For the focus group, both parents were encouraged to participate. We contacted the mothers according to the birth date of the child, starting with the youngest first. We did not contact mothers of children younger than one month. As a preparation we sent an e-mail with seven unfinished sentences (Table 1) to reflect upon – if possible between the two parents. The unfinished sentences were designed to give the parents an idea of what kind of questions we were going to discuss and give them an opportunity to form their own thoughts and opinions before entering the group.

**Table 1:** Unfinished sentences for the focus group interview and questions for the telephone interviews

**Focus group interview - unfinished sentences**	**Telephone interviews - questions**
1. When we agreed to let our child participate in the Calmette study the most important reason was…	1. What was your most important reason for participating in the study?
2. After signing the consent we thought…	2. How were you informed that your child was in the control group? When (counted in hours after the child’s birth) were you informed?
3. What worried us the most was…	3. Was it clear to you beforehand that it was a random process, whether your child was getting the vaccine or not? What do you think about that?
4. Immediately after we were told that our child was not going to be vaccinated, we thought…	4. What was your immediate reaction to being told that your child was not getting the vaccine?
5. What we are most delighted of now is…	5. What do you think now that you have had some time to consider the fact that your child is in the control group? Have you done anything on that note?
6. What we are most annoyed with now is…	6. What do you think of your continued participation in the study? Is there anything that has made you wonder?
7. What we still don’t understand is…	

We called the mothers of 40 infants who were consecutively enrolled in the Calmette study and achieved verbal contact with 34, 8 of which agreed to participate in the focus group. All of the mothers actually wanted to help, but either they had other plans at the time of the focus group or they said that it was too stressful to have to show up at a certain place on a certain time when the child was still so young. Two mothers cancelled a couple of days before the focus group and two mothers cancelled on the day of the focus group. The reasons were problems with breastfeeding, child going to the doctor, and that it was too stressful to have to show up. We tried to invite new mothers when the first two mothers had cancelled but it was not possible at such short notice. The focus group was therefore conducted with four mothers (M1 to M4), and two fathers (F1 and F2); the two fathers who did not attend were at work. The focus group took place approximately 5 weeks after delivery and randomization.

The interviewer (VZ) and two observers (SMP and GG) were present. The interviewer, a person unrelated to the Calmette study, followed a semi-structured form based on the seven unfinished sentences (Table 1). We addressed the questions of why the parents had decided to participate in the Calmette study, how they experienced the randomization process, how they had reacted towards being allocated to the control group, and finally their future involvement in the trial. The observers were present to take notes and observe non-verbal data. The focus group session lasted one hour and fifteen minutes. It was audio-recorded and afterwards a transcription was carried out by SMP.

### Telephone interviews

When recruiting for the focus group, it became clear that the majority of the mothers could not find time or energy to attend a focus group because of their child being so young. Some of them even asked why we could not just interview them over the phone which we consequently did. For practical reasons, we only talked with the mothers of the children, not the fathers. We called 29 of the 30 women who had been invited without attending the focus group, and we gave them at least one call. The one who was not contacted was excluded because she did not speak Danish. We achieved verbal contact with 23 mothers of which 19 were telephone interviewed (TM1 to TM19) including two of the four mothers who initially were supposed to participate in the focus group but who had cancelled. The four women who did not participate were all willing to complete the interview but were busy at the time of the phone call. Saturation was achieved after approximately 15 interviews, and having completed 19 interviews, we saw no need for contacting the last four women again.

The interview involved questions regarding demographical background and experiences with participation in the trial (Table 1). The interviews took place between 9 and 12 weeks after delivery and randomization.

### Demography

The age of the mothers in the study ranged from 25 to 42 and the fathers from 25 to 46. In two cases, the father was an unknown donor. All mothers, except one, had higher education. Ten of the mothers had children before the present one (range 1–8). The women had been in verbal contact with a member of the Calmette staff between 0 and 3 times before signing the consent form. The parents all lived in the metropolitan area of Copenhagen.

### Analysis of data

The focus group transcription was re-read several times by SMP in order to get familiarized with the data. Based on a preliminary inductive analysis of these data, a set of six open questions was developed to approach the most important topics as identified with the focus group. In this way the focus group inspired the subsequent telephone interviews. Notes made by SMP during the telephone interviews were transcribed afterwards and initially coded separately. Subsequently, we used inductive thematic analysis to identify important themes across the two data sets [19]. This meant searching for themes and patterns through the entire data corpus and the generated codes. For each identified theme, all the relevant data were found and grouped. Afterwards, the themes were gathered in mind maps to visually analyze how the different themes worked together, to fuse themes that covered the same aspect of the problem and to erase themes that were irrelevant. Data were organized in Microsoft Word.

**Figure 2:**
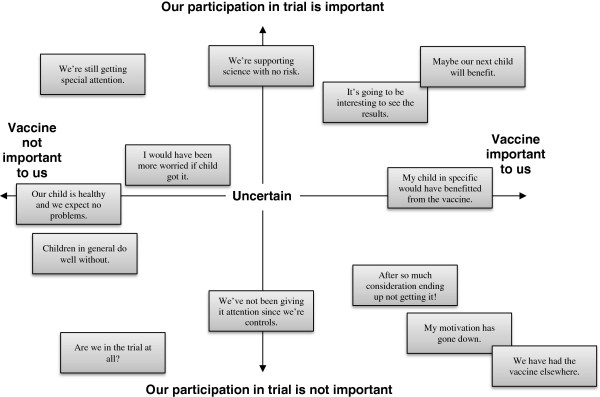
**Reactions from parents of newborn infants who were randomized to the control group.** The newborns participated in a vaccine trial and parents were not blinded to the allocation. Most of the parents were disappointed with the allocation; disappointment was followed by reactions that differed in degree and which could be positioned along the two continuities of this diagram.

Disappointment was an overarching reaction among participants. Further analysis revealed that the degree of the parents’ disappointment was connected with their positions on two continuities moving from ‘Vaccine not important to us’ to ‘Vaccine important to us’, and from ‘Our participation in trial is not important’ to ‘Our participation in trial is important’ (Figure 2). All analyses were performed by SMP and supervised by VZ.

### Ethics

The Calmette study is registered in EudraCT (https://eudract.ema.europa.eu/) with trial number 2010-021979-85 and was approved by the Danish National Committee on Health Research Ethics (approval number: H-3-2010-087). According to Danish law, interview studies are not considered biomedical research and therefore not subject to ethical review board approval, so the present study did not need another specific ethical clearance.

## Results

### Circumstances of the allocation process

Either a midwife or a member of the research staff told the parents that their child was going to be randomized, and the outcome was revealed immediately afterwards. In most cases of uncomplicated deliveries this took place in the delivery room and in a few cases the next day in the maternity ward. In general, the parents described the allocation process as non-stressful and said that it did not disturb the new family. When complications during labor affected either the child or the mother, parents were not approached until later, but the experience of the randomization process did not differ markedly for them.

Three families were familiar with the fact that their child had not received the vaccine but were uncertain as to whether their child was still a part of the Calmette study. Two of them had experienced post-partum bleeding and had been transferred for operation shortly after the child’s birth and thought that the personnel had postponed information about allocation, and that it eventually had been forgotten:

“*We somehow wondered if it had been recorded that he had been born*.” (TM9).

Of the 23 families we interviewed, 21 said they had understood that it was a random process that decided whether the child got the vaccine or not. Most said that they considered the random process as fair, and that they accepted that those allocated to the control group would not get the vaccine. Their acceptance was not necessarily based on an understanding of the rationale for randomizing but rather on an acceptance of randomization as a tool that researchers use:

“*It is a scientific trial, and this is the way you do these things.*” (F2).

The sentence “*That’s the way it is*” was repeated by many parents.Some parents made comments suggesting that they did not completely understand the importance of their child in the study when it was randomized to the control group, even if they said they understood.

“*(…) of course it was a shame that we weren’t really participating, but then again there were so many other things to think about.*” (TM11).

Two families had problems understanding the study design as a randomized controlled trial. One mother said that she had forgotten to inform the father of the child about the randomization part. He had not spoken with the research staff beforehand, but based on the mother’s explanation he had understood that agreeing to participate meant agreeing to let their child be vaccinated. The misunderstanding was not corrected until randomization was performed, and the child was allocated to the control group. Another mother said in the beginning of the interview that she was aware that her child might or might not get the vaccine, but later she wondered if her child did not get the vaccine because it was born during the night. She thought that the research group might have chosen to vaccinate only children born during daytime, when study staff was present.

### Disappointment or relief? – Immediate reactions to allocation

Parents of 21 out of 23 children expressed that they had hoped their child would receive the vaccine, especially because of the potential benefit of protection against atopic disease, but also a hope of protection against diseases in general was also mentioned. A few mentioned that protection against tuberculosis would be nice to have if their child was to travel later in life.

“*If you can have a child with an optimized health, it is fantastic, and it sounds like you could. That’s your hypothesis – and of course also that it didn’t imply any serious risks.*” (TM10).

The parents judged the risk of participation from very small to non-existing. The fact that the vaccine had been used for many years and that the risk of side effects was known to be small was an important reason for wanting to participate.

“*There weren’t any side effects, otherwise you wouldn’t dare. It is something that has been tried before, so there couldn’t be anything wrong in trying it.*” (T5).

The most common immediate reaction to the allocation was disappointment, but the degree of disappointment varied greatly. Most parents described pure disappointment whereas some felt ambivalent or had an immediate feeling of relief followed later by disappointment. Some were very disappointed*:*

“*My immediate* [reaction] *was a, a “crap” or “damn”…*” (M4).

This parent had spent a lot of time and energy discussing the allocation at home whereas other parents described their disappointment as a very short lasting emotion that was soon forgotten because of all the other events surrounding them as new parents.

Of the few women who did not express disappointment, one said that she felt relieved. Her child had been admitted to the neonatal department, and the worries that this might cause could well be an explanation.

By way of summing up, the parents’ diverse reactions to having their child allocated to the control group provided evidence for developing a model based on their positions on two continuities moving from ‘Vaccine not important to us’ to ‘Vaccine important to us’, and moving from ‘Our participation in trial is not important’ to ‘Our participation in trial is important’ (Figure 2).

### How important is the vaccine to us?

The parents who expressed frustration were often deeply engaged, either because they thought their child in particular would have benefitted from the vaccine, or because they had thoroughly considered and spent long hours deciding whether or not to participate.

Some of the parents were themselves affected by atopic diseases and were aware of the burden of the disease to which their children would be predisposed. These parents were disappointed by the allocation to the control group. This was expressed by a mother with atopic dermatitis who, despite having an immediate feeling of relief after allocation, later became aware of her disappointment:

“*Well, I kind of thought that, well in the beginning I thought: ‘Hmm, but that’s fine. Then he doesn’t have to be hurt by a needle and the scar and such, well, all such…’ And now I (…) think: ‘Hey, maybe I’m actually upset, because of the fact that it might have helped him, right?’ (…) so I would have liked that he got it, if it meant that he doesn’t have to suffer with the things I have now.*” (M1).

A parent of a child who had suffered from common colds expressed that she – despite immediate relief – now could not stop thinking that he might have avoided all that trouble if he had had the vaccine.

Parents who had spent time deciding whether or not to participate and hence invested emotions in the decision also expressed frustration. They had usually been in telephone contact with personnel from the research group more than once. Some had involved friends and family in order to decide, and one woman even described how her entire family had been against their participation.

“*I had a couple of friends who didn’t want it (…) I was at a family birthday party and everybody thought that… that we shouldn’t do it. (Laughs)*” (M2).

Parents who in this way had put time and energy into making the decision to participate described that the choice of participation in reality was an acceptance of the vaccine. Allocation to the control group was consequently felt to be a great waste of time and energy.

“*Definitely afterwards, also when you have made the decision to participate, and… considered all the things that could be negative and weighed it against the positive…*” (M1).

“*That’s it!*” (M2).

“*…then it’s a little… Hey, then you would have liked to… Now you have made all these decisions, so you would have liked to be, like, really participating.*” (M1).

In contrast, mothers who either said that they had not given the fact that they were in the control group much thought and had no special reason to want the vaccine, would often argue that their child probably would not have benefitted from the vaccine.

“*If he had been sick, I might have thought more about it and be even more annoyed about not being a part of it, because it might have prevented something*.” (TM14).

For some parents a smaller degree of disappointment also implied a neutral interest in following the results of the investigation as expressed:

“*Now we just want to follow the results of the investigations, you’re doing. We haven’t had any problems with her with diseases, such as asthma, and don’t expect to, so we’re just eager to follow the results.*” (TM16).

Only two parents expressed indifference concerning getting the vaccine, one arguing that she had only participated ‘to help science’, and the other saying that her primary reason for letting her child participate was the fact that participation could not have negative consequences; neither of them was disappointed.

### How important is my child to the trial?

Some parents expressed that they did not feel as important in the trial as they would have been if they had been allocated to the treatment group. They did not feel as a part of the study and they did not understand what their role was in the control group.

“*I would like to contribute, but I also think that the control group in this context is… It’s wrong to say less essential, because I know that there has to be a control group but… I’m also aware that there will be a lot of others, so there will be… others who can form the control group and (…) How come you don’t use, as en extra sort of control group, all the babies that… That are just ordinary, that are not in the study in any way.*” (M4).

“*The most interesting are those that participate by getting the vaccine.*” (TM10).

Others did not feel important in the trial either, but as they had not made a big investment in participation, they did not give their allocation much thought even though they had hoped for the intervention group.

“*(…) of course it was a shame that we weren’t really participating, but then again there were so many other things to think about*.” (TM11).

Others said that it made them feel good to be part of a trial and to contribute to science, meaning that they understood that they played a part in the trial even though they did not get the vaccine.

“*We contribute anyway. It means a lot that there is a control group.*” (TM6)*.*

Their disappointment was not so dominant, since they found participation meaningful.

“*Of course one hoped that he would be vaccinated, but it will also help in the future, if it is found that it was beneficial.*” (TM9).

### Conflict between the wish for intervention and the need for a control group

Three of the most disappointed families considered getting the vaccine elsewhere, and one of them had actually tried to get it:

“*But then I want to have the opportunity to give it to her via the private sector. And it looks like it is damn difficult. It makes me pissed. I think it is paternalistic!*” (M4).

The fact that they would not contribute to science if they got the vaccine elsewhere did not play an important role in this consideration. A family that had their child vaccinated at three days of age by their family doctor told a different story. The mother was not discontent with their allocation, because the parents had simply decided before the child’s birth that they wanted the vaccine no matter what, since the mother originated from a country where all children are vaccinated at birth. They had arranged with their family doctor that he would give the child the vaccine in case they were allocated to the control group. The mother said that she participated in the Calmette study partly to get the vaccine, but also because she had worked in research and knew that it was nice when people said yes to participation. She was not discontent with being allocated to the control group, because she knew that it was a possibility when participating in a trial.

### Adherence and future participation

None of the parents involved in the focus group or telephone interviews said that they would not participate in the follow-up planned in the Calmette study. Even the couple that had had their child vaccinated by their family doctor was happy to participate. One mother said that her motivation had decreased but that she would still participate as it was her civic duty. One of the participants of the focus group said that it had been a very good experience, emphasizing the importance of some kind of follow-up after randomization.

“*(…) I just think that it was really nice that there was a follow-up, about what you, what kind of considerations you can have afterwards. Because I think it can… It can also mean that you can have a, maybe a better talk with the parents who end up in this control group (…).*” (M3).

## Discussion

We found an almost universal expression of disappointment among parents of children who were randomized to the unblinded control group and had no intervention. However, the study also reflected general understanding and acceptance of the idea of randomization, and in some cases altruism. A few families had considered having their child vaccinated anyway, and one family actually did so. All families intended to participate in the study follow-up.

Why did the parents in this study express such good understanding of the random part of the RCT? Perhaps most importantly, all participants were well informed about the randomization process both in writing and orally before entering the trial. Moreover, the educational level of the participating families was high. A high level of education has been associated with better understanding of randomization [20,21], but not always so [22], and participants of all educational levels struggle with understanding it [23]. We asked the mothers participating in the focus group and the telephone interviews if they understood the random process. Maybe we would have found a poorer degree of understanding if we had asked the parents how allocation had been decided upon. Patients may not understand the study design, even though they feel well informed [2,20]. Finally, the parents in this study were given ample time to make up their minds about participation and the benefits of prevention of common diseases. Therefore, the circumstances were different from what parents have to face in acute and critical situations and this affects acceptance as well as understanding [2,22,24].

Most of the parents in this study had a preference towards one of the treatment arms and were therefore not in equipoise when entering. The parents describe how they perceive the risks to be small and the potential benefit to be large. Not surprisingly, the parents most willing to participate are those who believe the benefits of trial participation outweigh the risks [10,24-26]. It is noteworthy, however, that parents who are given time to think before the decision to enter their child in an RCT, as was the case in this study population, are likely to be more aware of the risks of participation [27,28].

It is argued that in a trial it is both possible and fair to get informed consent from parents/patients, even if they assume that the new treatment is better as long as they are well informed about the randomization and potential benefits and harms [24]. Many interventions tested in RCTs point in the direction that the benefits outweigh the harms, but that the existing evidence is still not strong enough to make the intervention ‘standard care’. In such situations, participants have good reason to prefer intervention to non-intervention. Nevertheless, if those randomized to the control group are significantly disappointed it may threaten participation in follow-ups, and it may also threaten the trial if control participants are able to get the intervention from elsewhere. If participants randomized to the control group are very disappointed it may also call in question whether the information given before consent was reasonably balanced. It may threaten the trust-relation between the community and the medical research establishment if interventions perceived as clearly beneficial are tested in placebo controlled RCTs.

A way of coping with parents’ disappointment when their child is randomized to the control group is to emphasize the important role of their child as a control and appeal to the parents’ altruistic motives for participation. Even though the benefits to the child seem to be of utmost importance to parents, it has previously been shown that parents also give altruism as a major motive for participation [29-31]. It is therefore important to make it clear to the parents that the control group is precisely as important for the trial as the intervention group.

**Figure 3:**
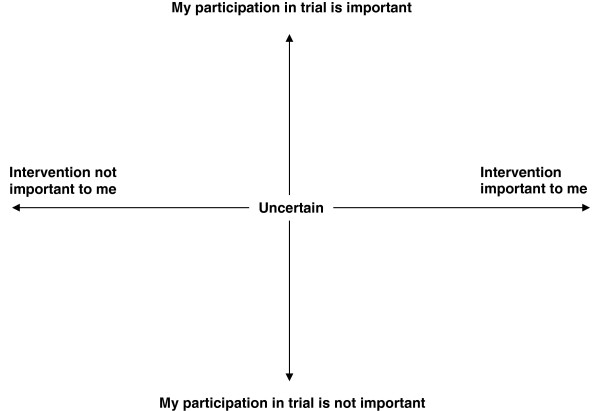
**Reaction to allocation to the control group in randomized clinical trial.** A tool designed to identify parents at risk of non-satisfaction, and thereby at risk of non-compliance or drop-out.

When informing parents that their child has been randomized to the control group it is possible to explore the parents’ understanding of the potential benefits and harms of the experimental intervention. The model we developed on the basis of the parents’ reaction to having their child allocated to the control group might, in a generalized version (Figure 3), serve as a tool for the use of researchers in order to identify parents at risk of non-satisfaction, and thereby risk of non-compliance or drop-out. It can be used in two ways: i) before the parents agree to participate in the trial, they are to be asked to place themselves in the diagram (Figure 3), trying to imagine that their child is allocated to the intervention group as well as to the control group; and ii) after allocation, the parents can look at the diagram and determine where they see themselves in the study. If the parents in either of the two situations place themselves extremely positively along the x-axis or extremely negatively along the y-axis, the researcher should review the trial information sheet with them in order to give them the best possible understanding of the trial. If the tool is used after allocation and the parents experience the trial information sheet as new information, the information given before informed consent may have been insufficient.

Such a tool needs further development and testing, and may also prove valuable for RCTs in the recruitment of consenting participants. The tool was developed too late for us to formally test it in the Calmette study, but the preliminary results of the present study made our team realize the importance of strengthening the feeling of identity and purpose in the control group by emphasizing to parents the essential role their child plays in gaining new insight. In the future, it would be interesting to evaluate the benefits of this tool by qualitative studies. By embedding such a tool in an RCT, it could be examined if study adherence in the control group can be improved.

As shown in this study and in the available literature, there is a lack of general knowledge about RCTs and what it means to participate in an RCT. This is of public interest since every citizen may be asked to participate in an RCT. One woman said that she participated because she considered it her ‘civic duty’. There could be more public focus on clinical trials. The topic could, for example, be part of the school curricula. First of all, it would be beneficial for the individual who would find her/himself to better able to decide, but it may also benefit science itself if participants were better educated.

### Strengths and limitations

This study has several strengths. Most importantly, it investigates the participants’ reactions after being allocated, whereas most of the literature has investigated the events leading up to allocation, namely the motives for participation before entering, the understanding of the randomization process, and problems with the informed consent. We set up a focus group and made 19 telephone interviews. We telephone-interviewed parents that could not participate in the focus group to keep selection bias as low as possible and we ended up having a satisfying rate of participation among the women we achieved verbal contact with. Through the telephone interviews we accomplished saturation. Finally, we interviewed the parents relatively shortly after randomization to minimize problems with recalling thoughts and reactions.

Our study has limitations. Regarding the study population, the parents investigated in this study may differ from the general population. They were well educated and all live in the metropolitan area of Copenhagen. Two of the mothers got pregnant using an anonymous donor. This may represent a selection bias in Rigshospitalet’s part of the Calmette study since, at the time of the interviews, 40% to 50% of the families approached by the Calmette study agreed to participate. Two fathers participated in the focus group, but we only talked to mothers in the telephone interviews. Finally, we only included the mothers who picked up their phone and had time for an interview immediately or at a later time the same week. Altogether, we obtained information from 23 out of the 40 mothers we initially tried to achieve verbal contact with, and they could differ from the 17 who did not participate. The mothers who were included might be the most disappointed who hence had interest in sharing their frustration.

We did not interview parents of children allocated to the intervention arm of the Calmette study, and therefore we are not able to determine whether the reactions of parents differ among the arms. We talked to parents of mostly healthy children; the findings in our study might have been different had we talked to parents of children who were ill [10]. Our study therefore addresses the attitudes and reactions of parents of healthy children rather than parents of children who were ill. Furthermore, our findings regarding parents of newborn children would not necessarily apply to parents of older children.

## Conclusions

This study identified an almost universal experience of disappointment among parents of newborns who were randomized to the control group in a study of BCG vaccination at birth. The understanding and acceptance of the randomization process were good, probably due to the comprehensive information material, the high level of education, and the ample time available for the information and consent process before birth. Based on the findings in this study, we suggest that exploring and correcting the parents’ understanding of the intervention, including uncertainty of beneficial effect, as well as potential harms, might lessen the feeling of disappointment. A useful way for research staff of dealing with the disappointment may be to emphasize the important role of the child in the control group and hence to appeal to the altruistic motives for participation.

## Abbreviations

RCT: Randomized Controlled Trial.

## Competing interests

The authors declare no competing interests.

## Authors’ contributions

SMP recruited participants, observed the focus group and performed the transcription, carried out the telephone interviews and the thematic analysis of all data, and drafted the manuscript. VZ participated in the design of the study, carried out the focus group, and supervised both the thematic analysis and the drafting of the manuscript. JK and LGS carried out the recruitment and allocation for the Calmette study and participated in the recruitment of participants for this study. GG conceived the study, participated in its design and coordination, observed the focus group and supervised the drafting of the manuscript. All authors revised the manuscript for important intellectual content and approved the final draft.

## Authors’ information

SMP and VZ are not involved in the Calmette study. JK, LGS and GG are part of the research group of the Calmette study.

## Supplementary Material

Additional file 1 Patient information sheet. Click here for file
