# Erratum to: Disappointment and adherence among parents of newborns allocated to the control group: a qualitative study of a randomized clinical trial

**DOI:** 10.1186/s13063-015-0616-2

**Published:** 2015-03-14

**Authors:** Sandra Meinich Petersen, Vibeke Zoffmann, Jesper Kjærgaard, Lone Graff Stensballe, Gorm Greisen

**Affiliations:** Department of Neonatology, the Danish National Hospital – “Rigshospitalet”, Copenhagen, Denmark; Steno Diabetes Center, Gentofte, Denmark; NKLMS, Oslo Universitetssykehus, Oslo, Norway; The Child and Adolescent Clinic, the Danish National Hospital - “Rigshospitalet”, Copenhagen, Denmark

## Erratum

After publication of this work [1], it has come to our attention that Lone Graff Stensballe’s surname was displayed incorrectly. The full list of authors has now been updated. We are publishing this erratum to update the author list, which is as follows:

Sandra Meinich Petersen, Vibeke Zoffmann, Jesper Kjærgaard, Lone Graff Stensballe and Gorm Greisen.
