# Supplementary material for: Disappointment and adherence among parents of newborns allocated to the control group: a qualitative study of a randomized clinical trial
Source: Trials. 2014 Apr 15;15:126. doi: 10.1186/1745-6215-15-126 (PMC4022324; doi:10.1186/1745-6215-15-126)
Supplement: Additional file 1 — Patient information sheet. [file 1745-6215-15-126-S1.docx]

The Calmette Study - Information for participants

Fact sheet concerning participation in a scientific study regarding whether vaccination can lower the general morbidity in Danish children: “BCG immunisation and morbidity among Danish children”

**Dear expecting parents**

We would like to ask you to consider letting the child you are expecting participate in a scientific study. Before you decide, we would like to inform you about the purpose of our study and why we are conducting it. Please read carefully the following fact sheet.

Within the next month, you will receive a call from a health worker who will tell you more about the study and answer any questions you might have. You can also choose to receive the information on the study in person instead. This can be arranged by phone. You are welcome to have a spouse or friend present during the conversation. If you decide to let your child participate in the study, please sign the consent form and mail it to us. Participation in the study is voluntary. You may withdraw your consent at any time without stating a particular reason. If you should choose to do so, it will not affect our treatment of your child or yourself.

**Study background**

The BCG vaccine against tuberculosis, also known as the Calmette vaccine, is one the oldest and most frequently used vaccines in the world. Studies from developing world countries suggest that the BCG vaccine has a positive effect on the immune system, resulting in fewer diseases and even increased chance of survival among the children who receive the vaccine. It has not been tested whether the BCG vaccine also has a beneficial effect on Danish children. We suspect it can protect against childhood infections and the common allergic diseases asthma and eczema.

**Study purpose**

The purpose of this study is to examine whether children who receive the BCG vaccine shortly after birth l experience less illness, less allergy, fewer hospital admissions and less use of medicine. In order to achieve dependable results, we need the participation of 4300 children. The children will be chosen randomly for vaccination within a week of birth or no vaccination. One half will receive the vaccine, the other half will not receive the vaccine.

All participating children will be followed up until two years of age.

**The course of the study**

As expecting parents you will receive a phone call from a health worker who will provide further information about the study and answer any questions you might have. The health worker will then ask you whether you would like to let your child participate or not. If you choose to participate, we will ask you to make a note of this in your pregnancy health record and put the sticker we have sent you on the pregnancy health record. During the phone conversation you will be asked some questions concerning asthma and allergies, smoking and pets in your family. When the baby is born you will be contacted by our staff. If you still wish to let your child participate, you will be asked to sign a consent form in case this has not already been done. Your child will subsequently be randomly assigned to either receiving the vaccine or not. The vaccination will take place immediately after the randomization.

When the child is three months old and again when it is 13 months, you will be called and interviewed about the birth and the health of your child. During the interview you will be invited for an examination of your child by a doctor at the hospital. At this examination your child will be examined, measured and weighed. Parents of children that are born prematurely will also receive a questionnaire concerning the development of the child when the child is 22 months.

**Benefits from the study**

The children who receive the BCG vaccination will be protected against tuberculosis. We are conducting the study because we *believe* the vaccine can also protect against infectious diseases, asthma and allergy. But we do not *know* - and that is the reason why it is necessary to carry out this scientific study. This study has been planned on such a large scale that we expect the results to be reliable.

**Side effects, risks, complications and inconveniences**

*It is common and does not require treatment that the vaccine may give rise to:*

- Redness, swelling and a sore on the skin where the vaccine has been given
- Swollen lymph glands approximately 4 weeks following vaccination
- A small, up to 5 mm wide skinscar where the vaccine has been given

The Calmette vaccine has been in use for almost 100 years and is a part of the childhood vaccination programme in more than 100 countries. Therefore the rare side effects are known:

*Uncommon side effects*

- Less than 1 in 100 children may experience headache, fever or suppurating sore
- Less than 1 in 1000 children may experience an abscess at the vaccination site
- Less than 1 in 1000 children may experience osteomyelitis (bone infection), infection of lymph glands or severe allergic reaction

Severe side effects are uncommon. Participation in the study is assessed to result in a very small risk that is counterbalanced by the possible significant advantages for the children due to protection against tuberculosis and a strengthened immune system.

If your child is vaccinated and you suspect side effects not described as common, please contact us. We will then advise and possibly examine your child and provide treatment if necessary. The children are insured under the Patient Insurance Act.

**Exclusion from study**

Children born before pregnancy week 32 or with a birth weight of less than 1000 grams, children with signs of immune deficiency and children requiring intensive care at the hospital are excluded from the study.

**Information regarding financing**

The study is planned by MD Lone Graff Stensballe and prof. Peter Aaby. The study is financed by Center for Vitamins and Vaccines (CVIVA) of the Danish National Research Foundation. There is no remuneration for participation.

**Access to study results**

The results of the study will be published regardless of whether the study shows an effect of the BCGvaccine or not. A summary of the results will be available at [www.calmette-studiet.dk](http://www.calmette-studiet.dk).

The study is terminated September 1^st^ 2015 and we expect to publish results from 2016.

**Conclusion**

We hope that this fact sheet has helped you understand what it means to participate in the study. We take the liberty of contacting you in any case by phone within the next month. This call will give you the opportunity to ask questions and we can tell you more about the study and the vaccine if you so wish. We also kindly ask you to read the enclosed sheet on “The rights of research participants in biomedical research projects”.

It you would like to know more about the study, you are most welcome to contact us.

**Contact and questions**

XXXXXXXXXXXXXXXXXXXXXXX
